# Amniotic membrane transplantation combined with cryotherapy vs. lamellar keratoplasty for medically refractory peripheral ulcerative keratitis: a retrospective cohort study

**DOI:** 10.3389/fmed.2026.1833378

**Published:** 2026-06-12

**Authors:** Jia Wan, Juan Bao, Cuiyu Wang, Ruiyao Gao, Kai Zhang, Yingxin Chen

**Affiliations:** Department of Ophthalmology, General Hospital of Northern Theater Command, Shenyang, China

**Keywords:** amniotic membrane transplantation, bridging therapy, cryotherapy, lamellar keratoplasty, peripheral ulcerative keratitis, refractory corneal ulcer

## Abstract

**Background:**

Medically refractory peripheral ulcerative keratitis (PUK) is a severe condition requiring urgent surgery. This study compares the clinical outcomes of amniotic membrane transplantation (AMT) combined with cryotherapy vs. lamellar keratoplasty (LKP) for this high-risk group.

**Methods:**

This retrospective cohort study evaluated patients with refractory PUK who underwent either AMT or LKP with a 12-month follow-up. Outcome measures included ulcer healing rate, best-corrected visual acuity (BCVA), epithelialization time, and recurrence rate.

**Results:**

A total of 58 patients (58 eyes) were included (AMT: *n* = 28; LKP: *n* = 30). Primary ulcer healing rates were comparable between the groups (AMT: 78.6%, LKP: 76.7%; *P* = 0.862). Both groups showed similar BCVA improvements up to 6 months postoperatively. However, the LKP group demonstrated significantly faster epithelialization (median 6 vs. 12 days; *P* < 0.05), superior BCVA at 12 months (*P* = 0.019), and a lower disease recurrence rate (10.0% vs. 32.1%; *P* = 0.038), although AMT demonstrated a superior safety profile with no procedure-related complications. Concurrent autoimmune diseases significantly prolonged epithelialization in both cohorts.

**Conclusions:**

LKP provides superior long-term optical/structural restoration and a lower recurrence rate, making it the definitive treatment for refractory PUK. However, AMT matches LKP in primary healing and mid-term efficacy. Amid global donor shortages, AMT serves as an invaluable, safe bridging therapy to stabilize the ocular surface and manage acute inflammation before definitive keratoplasty.

## 1 Introduction

Peripheral ulcerative keratitis (PUK) is a rare, vision-threatening inflammatory corneal disease, defined by the hallmark features of a corneal epithelial defect and progressive crescent-shaped peripheral stromal thinning. Clinically, patients present with severe ocular irritation, photophobia, and variable degrees of visual impairment (1). While optimized conventional systemic and topical immunosuppressive therapy remains the first-line management, a substantial subset of patients develop medically refractory PUK with ongoing stromal melting, requiring urgent surgical intervention to prevent corneal perforation and irreversible vision loss.

Commonly used procedures for non-perforated, medically refractory PUK include lamellar keratoplasty (LKP) and amniotic membrane transplantation (AMT), among others (2, 3). As a precise corneal reconstructive technique that preserves the host endothelium, LKP is well indicated for peripheral corneal lesions, with significantly lower risks of endophthalmitis and allograft rejection. Crescentic LKP yields excellent outcomes in small or crescentic peripheral corneal perforations and descemetoceles, reducing postoperative adverse events (4). By resecting diseased tissue while sparing normal stroma, LKP minimizes astigmatism and facilitates effective visual rehabilitation (5). However, the severe global shortage of corneal donor tissue remains a non-negligible limiting factor for LKP, especially in developing countries (6). Although the utilization of acellular, glycerol-preserved corneas has emerged to mitigate storage constraints and further reduce rejection risks, the absolute scarcity of graft material persists. AMT, acts as an effective biological shield that suppresses inflammation, and eliminates residual inflammatory foci, with the advantages of minimal invasiveness, no allograft rejection risk, and high accessibility amid global corneal donor shortage (7–9). However, there is a critical lack of head-to-head comparative studies evaluating the long-term efficacy and safety of AMT combined with cryotherapy vs. LKP for medically refractory PUK, failing to establish evidence-based surgical decision-making for this high-risk group.

To address this clinical imperative, this retrospective cohort study systematically compares the clinical outcomes of AMT combined with cryotherapy vs. LKP in patients with medically refractory PUK over a 12-month follow-up period. Furthermore, we aimed to evaluate the strategic utility of AMT in the context of global corneal donor shortages, specifically assessing its efficacy as a temporizing intervention to halt disease progression and optimize ocular surface conditions prior to definitive elective reconstructive surgeries.

## 2 Materials and methods

### 2.1 Study population

This retrospective study included patients diagnosed with PUK refractory to medical therapy at our hospital between January 2014 and June 2024. Patients were categorized into two groups based on the surgical procedure: the amniotic membrane transplantation combined with cryotherapy group (AMT group) and the lamellar keratoplasty group (LKP group). Based on a retrospective review of medical records, the surgical choice was primarily determined by patients' willing and the real-time availability of donor corneas. This study was approved by the Institutional Review Board of General Hospital of Northern Theater Command [Approval No. Y(2025)281] and adhered to the tenets of the Declaration of Helsinki.

All patients underwent a comprehensive preoperative systemic workup, including routine serological testing, infectious disease screening, and a detailed autoimmune profile. Rheumatology consultations were obtained to identify underlying systemic etiologies.

Inclusion Criteria: (1) Confirmed diagnosis of non-infectious PUK refractory to medical therapy. “Refractory to medical therapy” was characterized by the presence of any of the following despite optimal standard systemic and topical treatment (10, 11): (i) persistent or expanding ulceration and epithelial defects confirmed by slit-lamp and AS-OCT; (ii) deepening infiltration or progressive corneal thinning; or (iii) unremitting or worsening clinical symptoms (e.g., severe pain, photophobia, tearing); (2) Active ulceration characteristics: crescent-shaped peripheral corneal lesions spanning 2 to 10 clock hours, with a stromal depth ranging from 25% to 75% of the corneal thickness, and an ulcer width of 2–4 mm; (3) Patients who underwent primary AMT combined with cryotherapy or LKP; (4) Minimum follow-up period of 12 months; (5) Absence of other ocular pathologies affecting postoperative visual potential; (6) Age between 18 and 90 years.

Exclusion Criteria: (1) Presence of frank corneal perforation at the PUK site; (2) Ulceration involving the central optical zone; (3) Preexisting ocular comorbidities compromising visual acuity, such as glaucoma, macular degeneration, vitreous hemorrhage, retinal detachment, or optic atrophy; (4) Severe uncontrolled systemic immune diseases or autoimmune diseases in the active flare; (5) Pregnancy during the perioperative or follow-up period.

To minimize potential selection bias inherent to the retrospective design, strict inclusion and exclusion criteria were applied, and baseline characteristics were statistically evaluated to confirm comparability between the two groups.

### 2.2 Surgical methods

All surgical procedures were performed by the same experienced corneal specialist. In the LKP group, glycerol-preserved allografts from the Eye Bank of the General Hospital of Northern Theater Command were procured to minimize donor-related heterogeneity. These grafts were dehydrated in glycerol at 4 °C, stored for less than 3 months, and confirmed negative for infectious diseases. In the AMT group, freeze-dried amniotic membranes were supplied by Jiangxi Ruiji Bio-engineering Technology Co., Ltd. (China).

Routine preoperative preparation included eyelid margin cleaning and irrigation of the conjunctival sac and lacrimal passages for all patients. In the LKP group, miosis was induced preoperatively using pilocarpine nitrate eye drops (Zhenrui; Shandong Bausch & Lomb Freda Pharmaceutical Co., Ltd., China), administered every 5 min for 30 min (6 doses). No miotic agents were administered to the AMT group. Topical anesthesia with proparacaine hydrochloride (Alcaine; s.a. Alcon-Couvreur n.v.) was applied in both groups. The LKP group additionally received retrobulbar and peribulbar nerve blocks using lidocaine hydrochloride (Hubei Tiansheng Pharmaceutical Co., Ltd., China) to ensure adequate analgesia and akinesia.

For the AMT group, following topical anesthesia, the corneal ulcer and adjacent necrotic tissue were thoroughly debrided under an operating microscope to achieve a clean, smooth base. The excision extended 0.5–1.0 mm beyond the visible ulcer margins, with wider resection performed at the limbus to minimize recurrence risk. Cryotherapy was subsequently applied to the ulcer bed and margins at −60 °C for 10 s. A suitably sized piece of freeze-dried amniotic membrane was folded to fill the stromal defect and secured with 10-0 nylon interrupted sutures. The remaining amniotic membrane was then draped over the entire cornea and conjunctival surface as an overlay patch and anchored at the limbus with a 10-0 nylon continuous suture. The procedure concluded with the application of a bandage contact lens, tobramycin-dexamethasone ointment, and a pressure patch.

In the LKP group, following miosis induction and retrobulbar anesthesia, the extent of the lesion was marked using a corneal trephine. Lamellar dissection was performed to excise the diseased tissue layer by layer until a healthy stromal bed was reached; all excised tissue was submitted for pathological examination. The glycerol-preserved donor cornea, with both epithelium and endothelium removed, was trimmed into a “crescent” or “D-shaped” graft matching the dimensions and depth of the recipient bed. The graft was secured using 10-0 nylon interrupted sutures with knots buried in the stroma. Postoperatively, a bandage contact lens was inserted, followed by the administration of tobramycin-dexamethasone ointment and a pressure patch.

### 2.3 Postoperative management

Standard postoperative care for both groups included topical levofloxacin (4 times daily), bovine basic fibroblast growth factor (bFGF, 4 times daily), and tobramycin-dexamethasone ointment (once nightly). Upon complete epithelialization (confirmed by negative fluorescein staining), prednisolone acetate (4 times daily) and tacrolimus eye drops (twice daily) were added to enhance anti-inflammatory and anti-rejection control.

For AMT Group, the dosage of steroids and tacrolimus was tapered monthly based on clinical response, typically reducing to once daily before discontinuation. Sutures were generally removed 1–2 weeks postoperatively.

For LKP Group, patients with persistent epithelial defects lasting >1 week (delayed epithelial healing) underwent additional monolayer amniotic membrane transplantation. For patients with autoimmune diseases or high rejection risk, the tapering schedule for prednisolone acetate and tacrolimus was prolonged. Sutures were removed based on signs of loosening, significant neovascularization, or rejection. Additionally, patients with systemic autoimmune diseases received systemic immunomodulatory therapy until serological markers normalized, with monthly monitoring of liver and kidney function.

### 2.4 Outcome measures

Outcome measures included ulcer healing, best-corrected visual acuity (BCVA) at 2 weeks and 1, 3, 6, and 12 months postoperatively, and time to epithelialization. Additional parameters recorded included graft displacement or detachment, amniotic membrane dissolution time, corneal/graft transparency grading (12, 13), and corneal/graft neovascularization grading (14, 15). The rates of primary disease recurrence and surgery-related complications were monitored during hospitalization and follow-up.

Ulcer Healing (16) was defined as complete re-epithelialization with negative fluorescein staining, resolution of stromal infiltration, and stable corneal thickness; recurrence (17) referred to the re-emergence of inflammatory signs (e.g., infiltration, edema) or progressive thinning after initial resolution; and complications (17) encompassed all intraoperative and postoperative adverse events, including graft-related issues (rejection, melting, detachment), elevated intraocular pressure, and secondary infection.

### 2.5 Statistical analysis

Statistical analyses were performed using IBM SPSS Statistics 26.0. Continuous variables with a normal distribution (confirmed by the Shapiro–Wilk test), such as age and BCVA, are presented as mean ± standard deviation (SD) and were compared using the independent Student's *t*-test. Non-normally distributed continuous variables, specifically time to epithelialization, were analyzed using the Mann–Whitney *U*-test. Categorical variables—including sex, healing rate, corneal/graft transparency, neovascularization, and complication rates—were compared using the Chi-square test. Recurrence rates were analyzed using Kaplan–Meier survival curves and the log-rank test. A two-tailed *P*-value < 0.05 was considered statistically significant.

## 3 Results

### 3.1 Baseline characteristics

**Figure 1:**
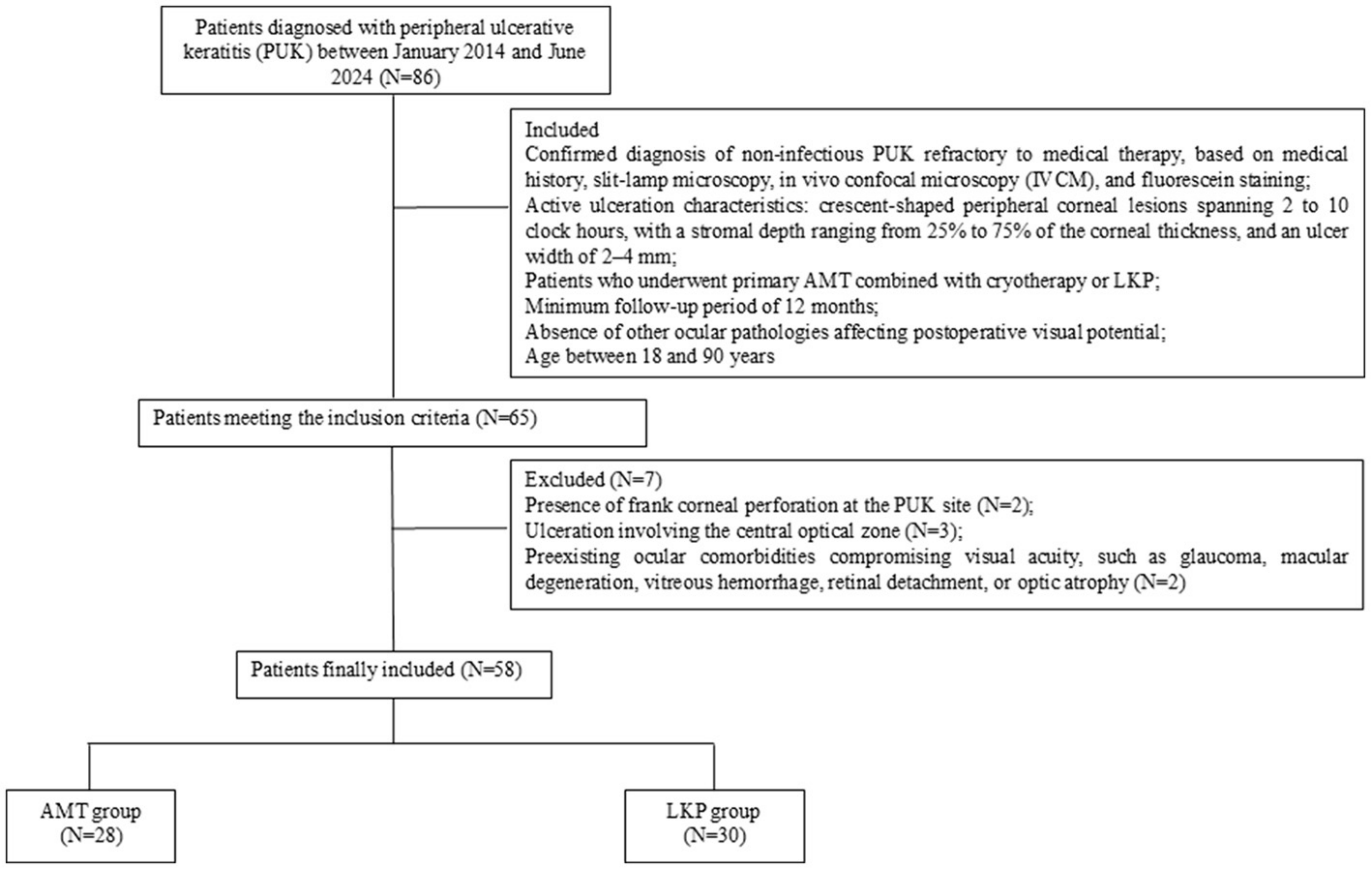
Flowchart. PUK, peripheral ulcerative keratitis; AMT, amniotic membrane transplantation; LKP, lamellar keratoplasty.

The patient enrollment process is illustrated in Figure 1. A total of 58 patients (58 eyes) were included: 28 in the AMT group and 30 in the LKP group. There were no statistically significant differences between the two groups regarding age (*P* > 0.999), gender distribution (*P* = 0.971), PUK, preoperative BCVA (*P* = 0.829), the presence of systemic autoimmune diseases (*P* = 0.637), or severity grading (*P* = 0.551), as detailed in Table 1.

**Table 1:** Baseline characteristics.

Variables	AMT group (*n* = 28)	LKP group (*n* = 30)	*P*-value
Age (years, mean ±*SD*)	58.0 ± 9.9	58.0 ± 11.2	>0.999
Male, *n* (%)	12 (42.9)	13 (43.3)	0.971
Preoperative BCVA (LogMAR)	0.71 ± 0.45	0.74 ± 0.46	0.829
Systemic autoimmune disease, *n* (%)	11 (39.3)	10 (33.3)	0.637
Severity grading, *n* (%)			
Mild	2 (7.1)	3 (10.0)	0.551
Moderate	15 (53.6)	12 (40.0)	
Severe	11 (39.3)	15 (50.0)	

### 3.2 Primary ulcer healing rate

**Figure 2:**
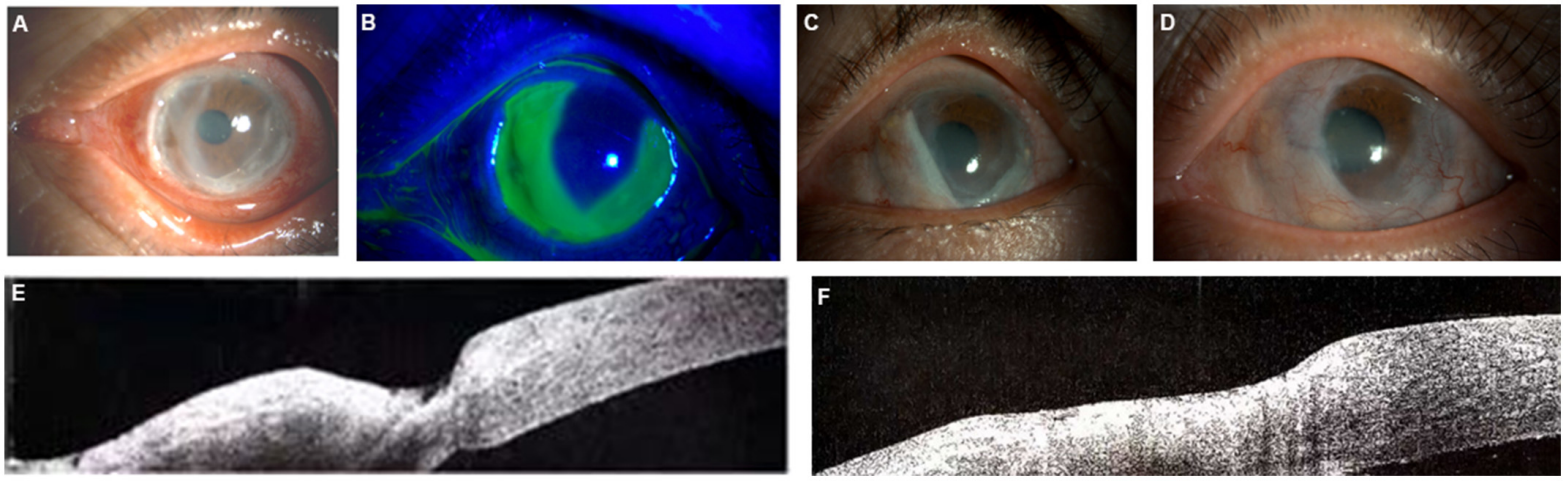
Representative case of successful AMT combined with cryotherapy. The patient was a 57-year-old female with a preoperative BCVA of 0.5 LogMAR. **(A, B)** Preoperative slit-lamp and fluorescein staining images showing a large, undermined crescentic epithelial defect from 2 to 12 o'clock. **(C)** One month postoperatively, demonstrating early healing with limbal neovascularization. **(D)** Twelve months postoperatively, showing stable conjunctivalization and vascularization (2 to 10 o'clock). **(E, F)** Preoperative and 12-month postoperative anterior segment optical coherence tomography (AS-OCT) revealing significant restoration of corneal thickness. The final BCVA improved to 0.4 LogMAR.

**Figure 3:**
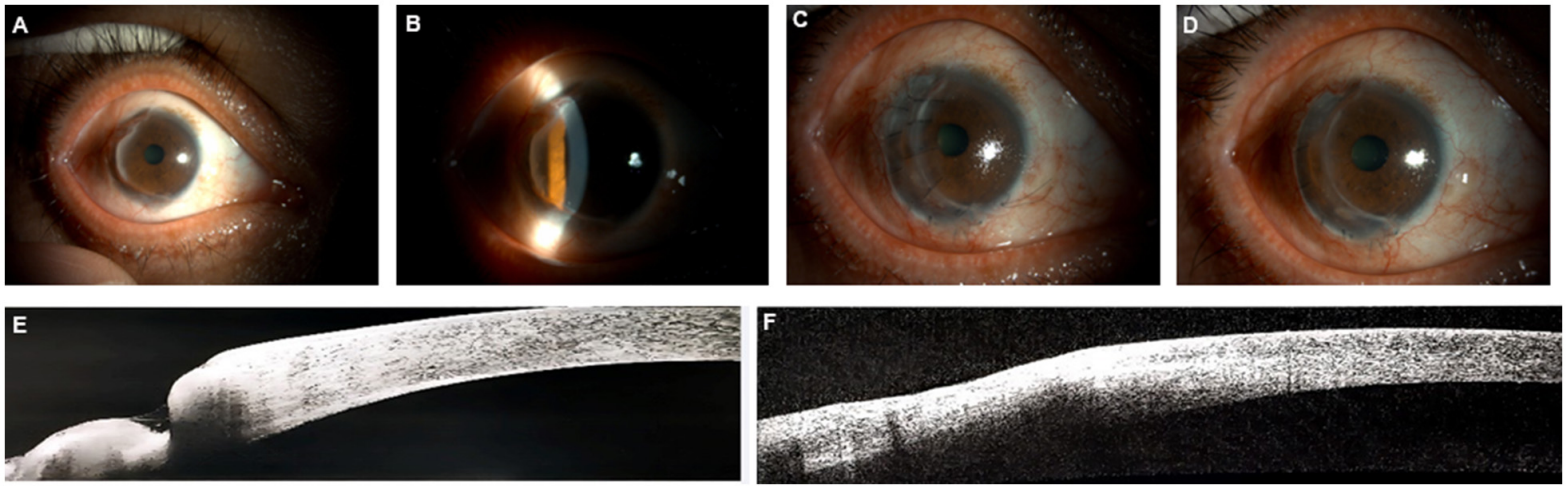
Representative case of successful LKP. The patient was a 57-year-old male with a preoperative BCVA of 0.1 LogMAR. **(A, B)** Preoperative slit-lamp images showing a furrow-like peripheral lesion from 5 to 12 o'clock. **(C)** One month postoperatively, showing a clear graft with excellent host-graft apposition and no edema. **(D)** Twelve months postoperatively, demonstrating sustained graft transparency following partial suture removal. **(E, F)** Preoperative and 12-month postoperative AS-OCT illustrating the anatomical restoration of corneal thickness and contour. The final BCVA remained stable at 0.1 LogMAR.

The primary ulcer healing rates were 78.6% (22/28) in the AMT group and 76.7% (23/30) in the LKP group. No statistically significant difference was observed between the groups (*P* = 0.862; Table 2). Representative cases showing successful outcomes are presented in Figures 2, 3. In the LKP group, six cases failed to heal after the primary surgery; five resolved with conservative treatment, and one achieved healing following subsequent AMT. In the AMT group, seven primary failures were observed. Of these, five healed with conservative therapy. Among the two cases refractory to conservative management, one resolved after undergoing LKP, while the other healed after the initial AMT but recurred 2 months later, eventually achieving resolution after a second AMT procedure.

**Table 2:** Postoperative outcomes comparison.

Variables	AMT group (*n* = 28)	LKP group (*n* = 30)	*P*-value
Ulcer healing rate, *n* (%)	22 (78.6)	23 (76.7)	0.862
Postoperative BCVA (LogMAR)			
2 weeks	0.91 ± 0.54	0.83 ± 0.46	0.575
1 month	0.71 ± 0.43	0.69 ± 0.34	0.848
3 months	0.66 ± 0.35	0.56 ± 0.29	0.228
6 months	0.62 ± 0.31	0.47 ± 0.27	0.055
12 months	0.60 ± 0.28	0.43 ± 0.23	0.019
Time to epithelialization [days, median (IQR)]	12 (10.3,13.0)	6 (5.0,7.0)	< 0.001
Recurrence rate, *n* (%)	9 (32.1)	3 (10.0)	0.038
Complication rate, *n* (%)	0	7 (23.3)	0.011
Graft rejection	0	4	
Transient IOP elevation	0	2	
Micro-perforation	0	2	

### 3.3 Postoperative BCVA

Postoperative BCVA improved over time in both groups. No significant differences in mean BCVA were found between the LKP and AMT groups at 2 weeks, 1 month, 3 months, or 6 months postoperatively (*P* > 0.05). However, at 12 months, the LKP group demonstrated significantly better visual outcomes compared to the AMT group (*P* = 0.019). Longitudinal intra-group analysis (Table 3) showed that BCVA in the AMT group remained stable, with no significant differences from the preoperative baseline at any follow-up point (all *P* > 0.05). Conversely, the LKP group exhibited significant visual improvement starting at 3 months postoperatively (*P* = 0.018), which was sustained and further enhanced through 12 months (*P* < 0.001).

**Table 3:** Intra-group comparison of BCVA (LogMAR) before and after surgery.

Time point	AMT group (*n* = 28)	*P*-value^a^	LKP group (*n* = 30)	*P*-value^b^
Preoperative	0.71 ± 0.45	–	0.74 ± 0.46	–
Postoperative 2 weeks	0.91 ± 0.54	0.110	0.83 ± 0.46	0.290
Postoperative 1 month	0.71 ± 0.43	1.000	0.69 ± 0.34	0.571
Postoperative 3 months	0.66 ± 0.35	0.568	0.56 ± 0.29	0.018
Postoperative 6 months	0.62 ± 0.31	0.308	0.47 ± 0.27	0.001
Postoperative 12 months	0.60 ± 0.28	0.193	0.43 ± 0.23	< 0.001

^a^*P*-values represent the statistical difference between each postoperative time point and the preoperative baseline within the AMT group.

^b^*P*-values represent the statistical difference between each postoperative time point and the preoperative baseline within the LKP group.

### 3.4 Time to epithelialization

The median time to epithelialization was 12 days (IQR: 10.3, 13.0) in the AMT group and 6 days (IQR: 5.0, 7.0) in the LKP group, representing a significant difference (*P* < 0.001; Table 2). Subgroup analysis revealed that the presence of systemic autoimmune disease significantly prolonged epithelial healing time compared to patients without autoimmune conditions in both groups (*P* < 0.05; Table 4).

**Table 4:** Comparison of time to epithelialization stratified by autoimmune disease status.

Groups	With autoimmune disease	Without autoimmune disease	*P*-value
LKP	7 (6.75, 8)	5 (4.25, 6)	0.001
AMT	13 (12, 15)	11 (10, 12)	0.037

### 3.5 Amniotic membrane dissolution

Amniotic membrane dissolution in the AMT group was completed between 7 and 12 days postoperatively. Specifically, complete dissolution was observed in three eyes at 7 days, 11 eyes at 8 days, four eyes at 9 days, seven eyes at 10 days, two eyes at 11 days, and one eye at 12 days.

### 3.6 Corneal and graft transparency

Postoperative transparency outcomes differed slightly between groups. In the LKP group (*n* = 30), transparency was classified as Grade 0 in 24 eyes, Grade one in five eyes, and Grade two in one eye. In the AMT group (*n* = 28), transparency was classified as Grade zero in 14 eyes, Grade one in 10 eyes, and Grade two in four eyes.

### 3.7 Neovascularization

Postoperative neovascularization was observed in four eyes (14.3%) in the AMT group (corneal neovascularization) and four eyes (13.3%) in the LKP group (graft neovascularization).

### 3.8 Postoperative complications and recurrence

Regarding surgical safety, the overall procedure-related complication rate was significantly higher in the LKP group (23.3%, 7/30) compared to the AMT group (0%, 0/28; *P* = 0.011, Table 2). No intraoperative or postoperative surgical complications occurred in the AMT group. Conversely, complications in the LKP group included transient intraocular pressure elevation (*n* = 2, resolved medically), intraoperative micro-perforation (*n* = 2, managed without aborting the procedure), and immunologic graft rejection (*n* = 4). Graft rejections were actively managed with intensive topical steroids, tacrolimus, and sub-brow corticosteroid injections; however, one case progressed to irreversible graft opacity and neovascularization.

**Figure 4:**
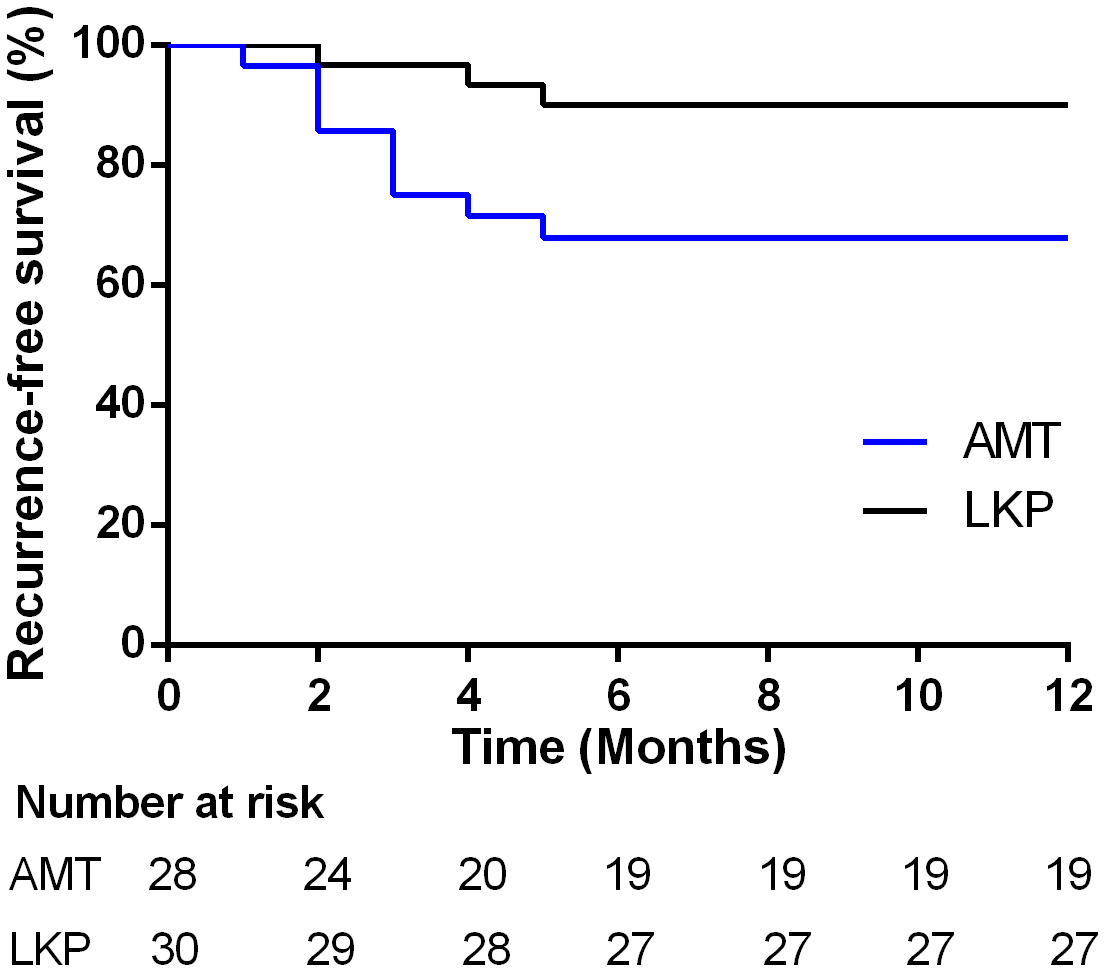
Kaplan–Meier analysis of recurrence-free survival. The LKP group demonstrated a significantly higher recurrence-free survival rate compared to the AMT group over the 12-month follow-up period (*P* = 0.038).

During the follow-up period, the overall recurrence rate was significantly higher in the AMT group (32.1%) compared to the LKP group (10.0%, *P* = 0.038; Table 2). In the AMT group, recurrences included seven *in situ* and two new lesions; these were managed with conservative treatment (*n* = 5), repeat AMT (*n* = 2), and conversion to LKP (*n* = 2, one of which yielded suboptimal vision after multiple AMT failures). Recurrences in the LKP group (three *in situ*) were successfully managed with AMT (*n* = 2) or conservative therapy (*n* = 1). Accordingly, Kaplan–Meier analysis demonstrated a significantly lower recurrence-free survival in the AMT group (Figure 4). Multivariate analysis adjusting for surgical method, sex, autoimmune disease status, and PUK severity confirmed that the difference in recurrence rates remained statistically significant, favoring LKP (Adjusted *OR* = 0.196, 95% *CI*: 0.042–0.920, *P* = 0.039).

## 4 Discussion

PUK is a severe, sight-threatening condition that often requires urgent surgical intervention when medical therapy fails. To our knowledge, this retrospective cohort study is among the first to provide a head-to-head comparison between AMT and LKP for medically refractory PUK. Our findings demonstrate that while LKP offers superior long-term visual rehabilitation and lower recurrence rates, AMT achieves equivalent primary ulcer healing and comparable mid-term visual outcomes. Importantly, these data strongly support a paradigm shift: AMT serves as a highly effective, safe temporizing or “bridging” intervention when donor corneas are unavailable or when systemic inflammation remains uncontrolled.

The most critical initial objective in treating refractory PUK is to halt active stromal melting and prevent perforation (1). Our results show that AMT and LKP are equally effective in achieving primary ulcer healing (78.6% vs. 76.7%, respectively). For patients with delayed healing, conservative management using bandage contact lenses and autologous serum eyedrops proved effective in providing a mechanical barrier and supplying essential epitheliotrophic factors (18, 19). Furthermore, BCVA remained comparable between the two groups up to 6 months postoperatively. Notably, the AMT group demonstrated a superior safety profile with zero procedure-related complications. This is a crucial finding, as it delineates a vital “window of opportunity.” By utilizing AMT—a widely accessible graft with no risk of allograft rejection—surgeons can successfully stabilize the ocular surface, buy essential time to optimize systemic immunosuppressive therapy, and wait for suitable donor tissue, all without compromising the eye's immediate tectonic integrity or short-term visual potential.

A key difference observed in the early postoperative phase was the time to epithelialization, which was significantly faster in the LKP group (median 6 days) compared to the AMT group (median 12 days). This is biologically plausible: LKP replaces the diseased stroma with a precisely tailored graft that provides an intact basement membrane scaffold, which synergistically interacts with the host's residual limbal and transient amplifying cells to accelerate epithelial repair and maintain homeostasis (5, 20, 21). Conversely, epithelialization in the AMT group relies entirely on host limbal cell migration across a debrided bed, a process further delayed by the transient microenvironmental disruption and early epithelial damage induced by adjunctive cryotherapy (22). Moreover, concurrent systemic autoimmune diseases significantly delayed epithelialization in both groups, likely due to an imbalance between matrix metalloproteinases (MMPs) and their tissue inhibitors (TIMPs) impairing limbal stem cell function (23). In this acute inflammatory phase, AMT acts as a biological shield, absorbing the localized immune attack until the systemic flare subsides. Maintaining this shield is vital; our observed median AM dissolution time of 12 days aligns with previous reports (24), and was likely prolonged by our use of highly adhesive freeze-dried AM and secure suturing techniques, which outperform sutureless alternatives (25, 26).

Despite the strategic utility of AMT, LKP remains the definitive procedure for long-term optical and structural restoration. As our 12-month data indicate, LKP yielded significantly better visual acuity. This divergence is anatomically driven: AM inherently possesses a variable spongy structure (27) and heals via opaque fibrotic scarring, whereas LKP provides a uniformly thick, structurally normal graft that maintains central visual axis stability, providing a biomechanical foundation for optimal long-term visual rehabilitation despite potential residual ametropia (28). Furthermore, LKP markedly lowered the recurrence rate (10.0% vs. 32.1%). This success hinges on the thorough excision of necrotic tissue—including adjacent involved conjunctiva and limbus—to clear target antigens and create a relatively “immune-privileged” bed (29). To further minimize graft rejection and neovascularization in these high-risk eyes, utilizing acellular glycerol-preserved corneas (30), combined with meticulous postoperative suture management (e.g., timely removal of loose sutures) (31) and tailored tapering of topical immunosuppressants (32), is essential.

The strength of this study lies in its real-world comparative design with well-balanced baselines, providing evidence that while LKP offers superior long-term visual and tectonic reconstruction, AMT serves as an indispensable, minimally invasive bridging therapy during global donor shortages. This study has certain limitations, primarily its retrospective design and the relatively small sample size inherent to the rarity of the disease. Future prospective, multicenter studies are warranted to validate these findings.

## 5 Conclusion

In conclusion, while LKP provides superior long-term visual outcomes and lower recurrence rates, AMT matches LKP in primary ulcer healing and short-to-mid-term efficacy. Amidst global donor shortages, AMT is a safe and invaluable bridging therapy that effectively stabilizes refractory PUK, allowing for delayed, optimized reconstructive keratoplasty.

## Data Availability

The original contributions presented in the study are included in the article/supplementary material, further inquiries can be directed to the corresponding author.
